# Urban-Rural Disparities for COVID-19: Evidence from 10 Countries and Areas in the Western Pacific

**DOI:** 10.34133/2021/9790275

**Published:** 2021-06-18

**Authors:** Minah Park, Jue Tao Lim, Lin Wang, Alex R. Cook, Borame L. Dickens

**Affiliations:** ^1^Saw Swee Hock School of Public Health, National Health Systems, National University of Singapore, Singapore; ^2^Department of Genetics, University of Cambridge, Cambridge CB2 3EH, UK; ^3^Mathematical Modelling of Infectious Diseases Unit, Institut Pasteur, UMR2000, CNRS, Paris 75015, France

## Abstract

**Background:**

Limited evidence on the effectiveness of various types of social distancing measures, from voluntary physical distancing to a community-wide quarantine, exists for the Western Pacific Region (WPR) which has large urban and rural populations.

**Methods:**

We estimated the time-varying reproduction number (*R*_*t*_) in a Bayesian framework using district-level mobility data provided by Facebook (i) to assess how various social distancing policies have contributed to the reduction in transmissibility of SARS-COV-2 and (ii) to examine within-country variations in behavioural responses, quantified by reductions in mobility, for urban and rural areas.

**Results:**

Social distancing measures were largely effective in reducing transmissibility, with *R*_*t*_ estimates decreased to around the threshold of 1. Within-country analysis showed substantial variation in public compliance across regions. Reductions in mobility were significantly lower in rural and remote areas than in urban areas and metropolitan cities (*p* < 0.001) which had the same scale of social distancing orders in place.

**Conclusions:**

Our findings provide empirical evidence that public compliance and consequent intervention effectiveness differ between urban and rural areas in the WPR. Further work is required to ascertain the factors affecting these differing behavioural responses, which can assist in policy-making efforts and increase public compliance in rural areas where populations are older and have poorer access to healthcare.

## 1. Introduction

Targeted nonpharmaceutical interventions, including the quarantine of potentially infected individuals and isolation of confirmed positive cases, form the basis for control of respiratory infectious disease epidemics in the absence of widely available vaccines and prophylactics. When diagnosed cases are responsible for few transmission events, as has been the case during the coronavirus disease 2019 (COVID-19) pandemic, untargeted interventions that affect the wider community may be needed. Social distancing measures including lockdowns have been used by many countries to limit the number of contact events between individuals and thereby reduce transmission [1–3].

While it has affected all six WHO regions, countries and areas in the Western Pacific Region (hereafter, “WPRs”) including Japan (Jan 15); South Korea (Jan 20); and Singapore (Jan 23) were among the first to report confirmed cases of COVID-19 outside mainland China [4]. With limited time to prepare for the emergence of the novel coronavirus, due to their proximity to the initial epicentre, WPRs quickly implemented strict social distancing measures to delay spread and expand health system capacities. Social distancing measures including stay-at-home orders have shown to be highly effective in reducing and slowing the transmission of COVID-19, which is mainly spread through respiratory droplets, by limiting close contacts between susceptible and infected individuals who may not be aware of their infection [5]. The ability to control their national outbreaks has been remarkably heterogeneous with many WPRs undergoing second or third epidemic waves [6–9] as of November 2020, occurring after the easing of movement restrictions as a response to increasing concerns on rising socioeconomic costs and adverse mental health impacts [10, 11].

Notably, many WPRs have large urban and rural populations with older rural populations more likely to have higher rates of underlying chronic disease and risk of complications if infected and poorer access to healthcare [12, 13]. To date, limited information exists on whether COVID-19 social distancing measures are equally effective in urban and rural regions where the exploration of any disparities can assist in appropriate policy-making efforts which are tailored. Utilising mobility data [14], we evaluate the effectiveness of social distancing measures in reducing transmission throughout the pandemic period across urban and rural provinces in the WPRs. The region is diverse in terms of economic status, cultures, government perceptions, and adherence to authority and control measures, which allows us to assess social distancing efficacy across a wide range of settings.

In this study, we (i) compare changes to mobility over the first six months of the pandemic (March to August 2020) across 10 WPRs including Australia; Hong Kong, SAR China (hereby called Hong Kong); Japan; Malaysia; New Zealand; the Philippines; Singapore; South Korea; Taiwan, China (hereby called Taiwan), and Vietnam; (ii) explore associations between mobility and transmission of SARS-CoV-2 by inferring the effective reproduction number (*R*_*t*_); and lastly (iii) examine within-country variations in behavioural responses to government policies and regulations, quantified by reductions in mobility, in urban versus rural areas, and how that has contributed to reductions in transmission in the aforementioned WPRs and areas.

## 2. Methods

### 2.1. Data Source and Description

We used two high-resolution human mobility datasets from Facebook that included changes in movement before and during the first six months of the COVID-19 pandemic (March through August 2020), in which varying levels of social distancing measures were in place. Taken from the Database of Global Administrative Areas (GADM), the spatial unit for both datasets was an administrative district or a village for a given city or province, respectively. Changes in mobility refer to a proportional increase or decrease, computed from the number of trips made from one district to another relative to a baseline period, which is (i) February 2020 (Facebook Movement Range Maps) or (ii) a set period of 45 days prior to the day that the data were generated (Facebook's Data for Good Initiative).

The first mobility data set was publicly available and was extracted from Facebook Movement Range Maps [15]. This anonymized, aggregated data included (i) the daily average mobility changes for each district *i* relative to a baseline of February 2020, which predates social distancing measures implemented in most WPRs, and (ii) the proportion of individuals staying within a single district during an entire day (i.e., stay-at-home), for each of predefined spatial units in the WPRs of interest. This dataset was used for all countries but Singapore, for which data was not available at the time of the analysis. The second dataset was obtained through our collaboration with Facebook's Data for Good initiative. The data included more detailed human mobility datasets with granular geospatial resolution at every 8 hours during baseline and pandemic periods. For each pair of districts *i* and *j*, the dataset included (i) the total number of users moving from district *i* to *j*, and vice versa, thereby allowing us to explore how people moved around within and across different districts (and cities), and (ii) other geospatial information including the latitude and longitude of the corresponding district. Although this second dataset allowed us to explore not only within-city but also between-city movement, we focused on changes in the “within-city” movement as it more accurately reflects the impact of social distancing measures (e.g., closing of schools, restaurants, and other entertainment venues) on daily lives. We used the number of trips made between these two districts during the baseline period (*N*_Base,*i*_) and the intervention period (*N*_Int,*i*_) to calculate changes in the movement for a given district and for each day: (*N*_Base,*i*_ − *N*_Int,*i*_)/(*N*_Base,*i*_).

We used ArcGIS Desktop by ESRI (Version 10.8) to match an administrative district with a corresponding city or province based on the unique polygon ID given to each district in the Facebook data. Epidemiological data and information on social distancing policies for each country and area were extracted from multiple sources including government websites (e.g., Ministry of Health, Center for Disease Control) and local media [4, 6–9, 16–20].

### 2.2. Data Analysis

#### 2.2.1. Mobility Trends in Response to Social Distancing Measures

We performed a descriptive analysis to explore overall trends in within-city (i.e., between-districts) movement for each city and country in the WPRs and how the public responded to mandatory or voluntary social distancing policies implemented during the COVID-19 pandemic within and across WPRs. Specifically, we compared the changes in mobility during the period in which strict social distancing or community-wide stay-at-home orders (i.e., lockdown) were in place in urban and rural areas for each country. For within-country comparisons, we used the level of urbanization reported from each country's government website to classify states and provinces into urban and rural areas [21, 22]. We then conducted Welch's *t*-test to compare the mean of mobility reduction in urban and rural areas.

#### 2.2.2. Estimation of the Time-Varying Effective Reproduction Number (*R*_*t*_)

We used a novel Bayesian estimation framework, known as the regression augmented time-varying reproduction number, which is able to infer both the time-varying reproduction number as well as the effects of other covariates on the said epidemic quantity [23].

We defined the time-varying reproduction *R*_*t*_ following Cori et al. 2013 [24], as the ratio of the number of new secondary cases reported in a given spatial unit on time *t* and the total infection potential across all infected individuals at time *t* as *Λ*_*t*_. In our proposed framework, we also allow *R*_*t*_ to be affected by two other covariates, namely, the proportion of users staying put at time *t*, *U*_*t*_, as well as the proportional change from baseline mobility at time *t*, *P*_*t*_. The infection potential across time is parameterized by the probability mass of the serial interval *w*_*S*_, lasting *s*  time units, and is defined as

(1) $$\begin{array}{c} \Lambda_{\text{t}}\left({\text{w}}_{\text{s}}\right)=\overset{\text{t}}{\underset{\text{s}=1\;}{\sum }}{\text{I}}_{\text{t}-\text{s}}{\text{w}}_{\text{s}}. \end{array}$$

Given the serial interval distribution *w*_*s*_, the total number of past incident cases *I*_*o*:*t*−1_ and the time-varying reproduction number *R*_*t*_ at time t, we have by definition the expected number of incident cases, namely,

(2) $$\begin{array}{c} \text{E}\left({\text{I}}_{\text{t}}\;|\;{\text{I}}_{\text{o}:\text{t}-1},{\text{w}}_{\text{s}},{\text{R}}_{\text{t}},{\text{X}}_{\text{t}}\right)={\text{R}}_{\text{t}}\Lambda_{\text{t}}. \end{array}$$

We assume that the number of incident cases follows a data generating process parameterized by the conventional Poisson distribution at time step *t*. As a result, we have the following conditional probability function of observing *I*_*t*_ cases at time *t*,

(3) $$\begin{array}{c} \left(I_{t}\;|\;I_{o:t-1},w_{s},R_{t},X_{t}\right)∼\text{Po}\left(R_{t}\Lambda_{t}\right). \end{array}$$

We further allowed the time-varying reproduction number to depend on two other covariates, namely, the proportion of users staying put at time *t*, *U*_*t*_, as well as the proportional change from baseline mobility at time *t*, *P*_*t*_, nested within a linear regression framework for parsimony. These are weighted by the kernel *K*, parameterizing the effect of time decay of exogenous measures on the time-varying reproduction number, as below

(4) $$\begin{array}{c} {\text{R}}_{\text{t}}=\beta_{0}+\beta_{1}\overset{\text{t}}{\underset{\text{L}=1\;}{\sum }}{\text{K}}_{\text{t}-\text{L}}{\text{U}}_{\text{t}-\text{L}}+\beta_{2}\overset{\text{t}}{\underset{\text{L}=1\;}{\sum }}{\text{K}}_{\text{t}-\text{L}}{\text{P}}_{\text{t}-\text{L}}+\epsilon_{\text{t}}, \end{array}$$

where *β*_0_ is an intercept term, *β*_1_, *β*_2_ the coefficients of interest, which can be interpreted as the expected change in *R*_*t*_ given a one-unit change in *U*_*t*−*L*_ or *P*_*t*−*L*_  over the interval [*t* − *L* : *t*]. The white noise term *ϵ*_*t*_ ~ *N*(0, *σ*_*ϵ*_^2^) is set to be an independent Gaussian with variance *σ*_*ϵ*_^2^ assuming that infectiousness and time to symptom onset varies in time among infected individuals.

#### 2.2.3. Implementation

The estimation of all parameters of interest [**R**_**t**_, *β*_1_, *β*_2_] are nested within a Markov Chain Monte Carlo (MCMC) framework described previously [23]. We ran the MCMC procedure on the finest possible spatial resolution for six WPRs according to case data availability in WPRs with a total of 10,000 iterations for each subregion. Gewecke convergence diagnostic checks and visual inspection of trace plots were used to determine convergence. The incubation period and serial interval used in this study were taken from published literature [25].

All statistical analyses were conducted in R version 4.0.2 (R Foundation for Statistical Computing, Vienna, Austria).

## 3. Results

**Figure 1:**
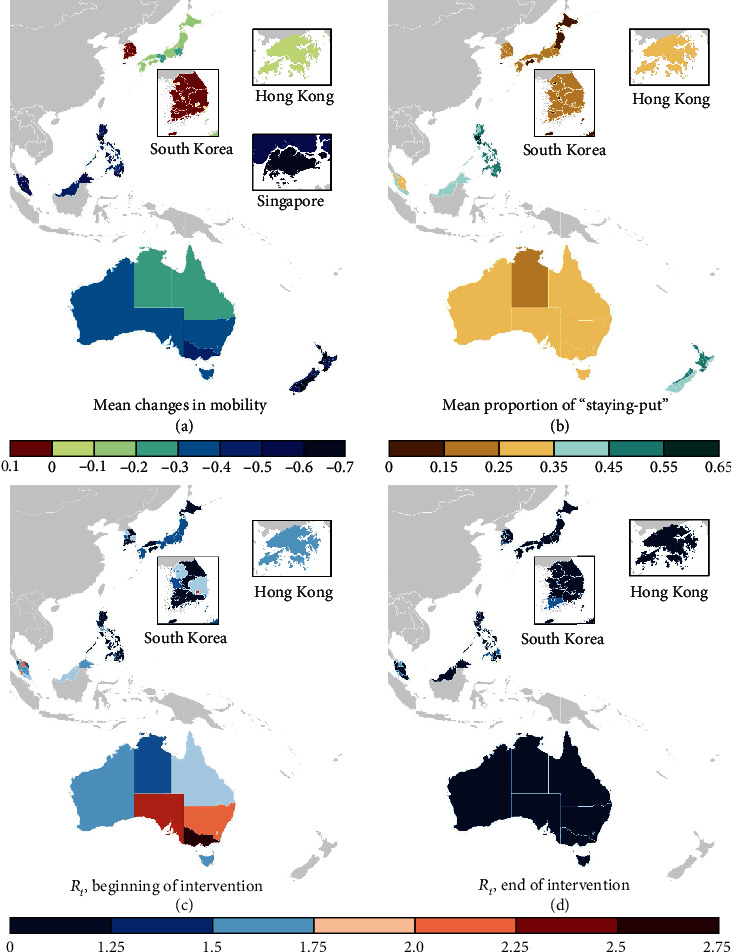
Changes in mobility and the time-varying effective reproduction number across countries and areas in the WRP. (a) Mean changes in mobility during which social distancing measures were in place (e.g., - 0.3 = a 30% reduction in mobility compared to February 2020). (b) Mean proportion of individuals staying put within a single district during the intervention. (c) The expected time-varying reproduction number at the beginning of the intervention. (d) The expected time-varying reproduction number at the end of the intervention.

We examined the trends in mobility and transmissibility of COVID-19 over the six months in the early stage of the pandemic (March 1 to August 31, 2020) to exclude any potential effects of other factors that could affect behavioural responses and public adherence to social distancing measures (e.g., vaccination or “pandemic fatigue” due to prolonged lockdown). Our findings show that initial social distancing measures implemented by most WPRs during the COVID-19 pandemic have been largely effective in reducing mobility (Figures 1(a) and 1(b)) and transmissibility (Figures 1(c) and 1(d)), with the effective reproduction number reduced in 61 of 70 subregions included in the study. Please see Supplementary Figures for the timing of intervention periods in each country.

### 3.1. Reductions in Relative Mobility during the COVID-19 Pandemic: Across 10 WPRs

Public compliance with social distancing measures, measured by the reduction of movement in the population compared to the baseline period, varied across WPRs. The overall reduction in mobility was greater in WPRs with tighter regulations (i.e., mandatory stay-at-home orders) than in WPRs with less strict, voluntary social distancing measures.

The median reduction in mobility was the largest in Singapore (64%) between Apr 7 and June 1, 2020, during which a stay-at-home order (“Circuit Breaker”) was in place (Supplementary Figure 7B). New Zealand (60%) also showed a significant overall reduction in mobility ranging from 54% for Gisborne to 67% in Auckland City, between Mar 26 and Apr 28, 2020, during which a nationwide lockdown was imposed (Supplementary Figure 7).

The WPRs with voluntary social distancing orders saw relatively smaller reductions in the movement of the population (Figure 1(a)). In South Korea, a large variation in mobility was observed across regions between February 29 and May 5, 2020, when the social distancing order was tightened to Level 2, involving the closing of high-risk facilities including nightclubs, karaoke bars, and gyms. While the relative mobility was reduced by up to 8% in Busan metropolitan city, it increased by up to 8% in Jeonbuk province at the same time. All remaining WPRs exhibited substantial reductions as presented in Figure 1(a) and Table 1.

As for the proportion of individuals who stayed within a single district, there was no significant difference across subregions (Figure 1(b)) and it was not correlated with the expected reproduction number.

### 3.2. Correlation between Mobility and the Effective Reproduction Number (*R*_*t*_)

We estimated the time-varying effective reproduction number for six WPRs, for which city- or state-level case data were available at the time of the analysis.

As seen in Table 1, community-wide control measures including social distancing or stay-at-home orders led to reductions in both mobility and transmissibility in all key cities and states. The median mobility reduction ranged from 2% in Hong Kong to 69% in Manila, where social distancing and community-wide stay-at-home orders were imposed, respectively. The time-varying effective reproduction number was also lowered to below or close to the threshold of 1 in most WPRs, ranging from 0.80 (0.72–0.89) for Tokyo to 1.19 (1.07–1.32) for Victoria, by the end of the intervention period.

**Figure 2:**
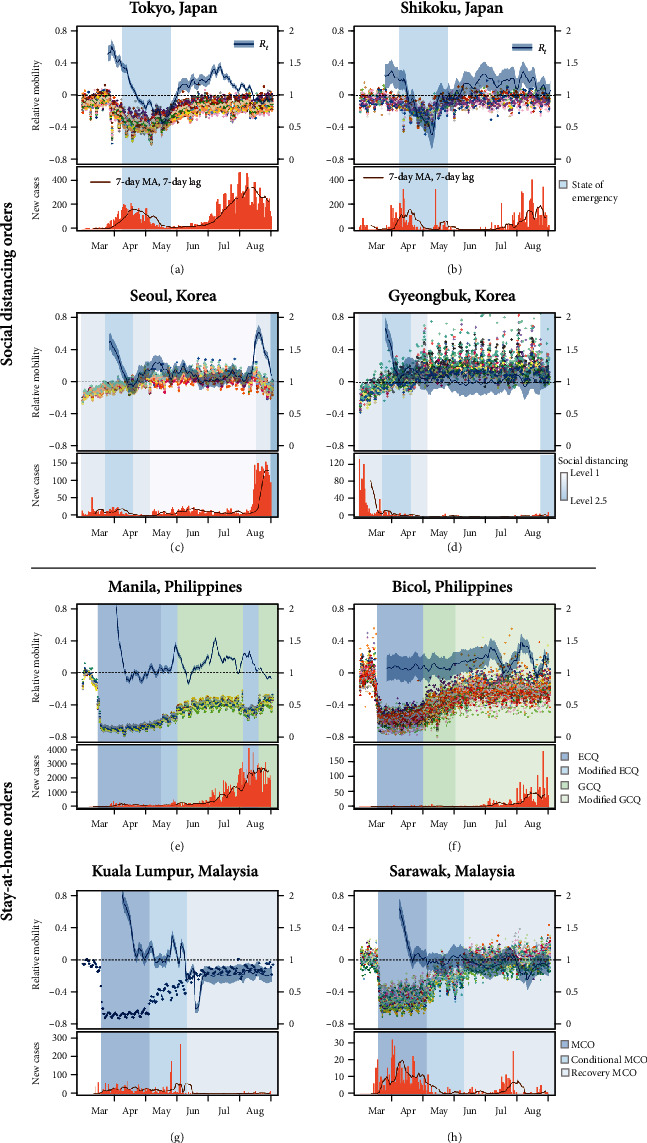
Changes in mobility and the time-varying reproduction number (top) and the number of new daily reported cases (bottom) between March 1 and August 31, 2020, by the region. Panels (a), (c), (e), and (g) show urban areas, which are the capital cities of Japan, Korea, the Philippines, and Malaysia, respectively. Panels (b), (d), (f), and (h) show corresponding less urbanized areas in the same countries. Each point indicates daily movement change relative to a baseline of February 2020, and each colour represents an administrative district for each city. The background shading indicates periods of intervention of differing social distancing and lockdown levels for each region, with darker colours indicating tighter regulations.

Our analysis shows that the expected effective reproduction number, *R*_*t*_, closely follows changes in mobility (Figure 2). Here, we compare trends in mobility and transmission in WPRs that have implemented voluntary social distancing measures (Japan and South Korea—Panel A, B, E, and F) and that with mandatory stay-at-home orders (The Philippines and Malaysia—Panel C, D, G, and H).

A substantial reduction in the *R*_*t*_ from a maximum of 1.77 (95% CrI: 1.66–1.88) to the lowest 0.57 (95% CrI: 0.52–0.62), along with a decrease in the relative mobility, was observed in Tokyo where a “State of Emergency” was in force from April 8 to May 25, 2020. Following the easing of movement restrictions, however, mobility bounced back to prepandemic period levels and the expected *R*_*t*_ sharply increased to above the threshold of 1, which was then followed by a resurgence of infections (Figure 2(a)). This pattern was also observed in the Shikoku prefecture of Japan, the most rural of Japan's four major islands, where the implementation and ease of social distancing policies were followed by a decrease and increase in the *R*_*t*_, respectively (Figure 2(e)). Similarly, another country that has adopted a moderate level of voluntary social distancing measure was South Korea, which saw a sharp drop in the expected *R*_*t*_ during the period in which the social distancing level was further tightened from March 21 to April 19, 2020. Consistent with the overall mobility which remained relatively stable for the next four months with relaxed measures, the expected reproduction number stayed between 1 and 1.5 in both urban and rural areas. The expected reproduction number went back up, however, to around 2 in Seoul, the most populous city in the country, leading to the introduction of tightest movement restrictions with a resurge in the number of new cases in August 2020.

In Malaysia, strict movement restriction policies imposed at around the same time as the Philippines have led to a substantial reduction in both mobility and transmission (Figures 2(g) and 2(h)). The nationwide lockdown remained in place throughout the early phase of the outbreak in both Kuala Lumpur and Sarawak, reducing the movement up to 72% and 70%, respectively, and the expected *R*_*t*_ to around 1. While While mobility continued to increase as the country gradually lifted the restructions throughout the recovery period, the reproduction number remained zero in both regions.

### 3.3. Reductions in Relative Mobility and Transmissibility: Within-Country Analysis

**Figure 3:**
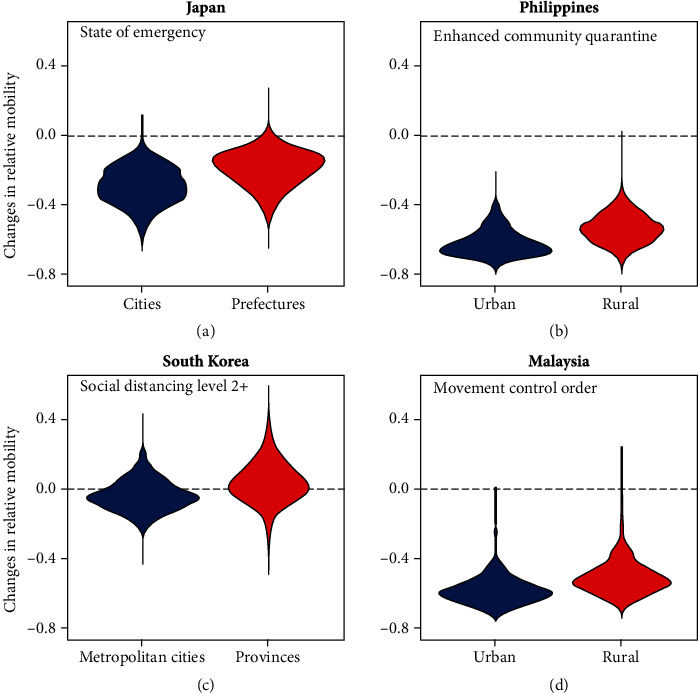
Changes in mobility split by urban and rural groupings for countries with social distancing (Panels (a) and (c)) and stay-at-home orders (Panels (b) and (d)).

As shown in Figure 3, there was a statistically significant difference (*p* < 0.001, Welch's *t*-test) in the mean changes in mobility between urban and rural areas during the period in which social distancing or stay-at-home orders were imposed. In all 10 WPRs included in the study, reductions in mobility were greater in urban areas (i.e., metropolitan cities or states with higher than a national average level of urbanization) than in rural areas (Figure 3). In particular, a substantial reduction in relative mobility of 62% and 58% was seen in the urban areas of the Philippines and Malaysia, respectively.

The only country which saw an increase in the overall movement was South Korea with a 5% (range: 2%–9%) increase in rural areas (provinces) during which social distancing measures of Level 2 or above were in place.

Similarly, higher reductions in transmissibility were observed in metropolitan cities and urban areas compared to rural provinces. Out of a total of 70 areas included in the analysis, nine saw an increased effective number during the intervention period, of which seven were less urbanized provinces. Within each country, the greatest reductions in the expected reproduction number were observed in metropolitan cities and urban areas including Manila (57%) in the Philippines, Daegu in South Korea (55%), Johor (50%) and Kuala Lumpur (44%) in Malaysia, and Tokyo (45%) in Japan.

## 4. Discussion

Over the past six months of the COVID-19 pandemic, WPRs have experienced the emergence and resurgence of infections during which social distancing measures were tightened or eased depending on the epidemic situation and trajectory. Having experienced previous outbreaks of novel coronaviruses, such as SARS in 2003 (Hong Kong; Singapore; Taiwan) and MERS-CoV in 2015 (South Korea), many of these WPRs were able to quickly implement control measures and successfully managed to contain and slow spread in the early phase of the epidemic. Yet, we found that WPRs had substantial within-country variations with greater reductions observed in mobility and transmissibility in urban areas when compared to rural or more remote provinces.

Overall, the implementation of social distancing measures contributed to the mitigation of spread in all WPRs where the expected reproduction number of SARS-CoV-2 fell to below or close to the threshold of 1 (or decreased by up to 67%) by the end of the intervention period and the epidemic curve flattened shortly after. Similarly, we have shown that easing movement restrictions could potentially lead to a resurgence of infections. In Hong Kong, for instance, as mobility increased by more than 20% compared to the baseline with the easing of restrictions in May and June 2020, the expected **R**_**t**_ gradually increased to as high as 1.55 (95% CrI: 1.41–1.68) which then potentially led to the second wave in July through to August (Supplementary Figure 1A). The same pattern was observed in Tokyo, Seoul, and the Philippines (Figure 3), where increased mobility following the ease of restrictions contributed to a sharp rise in the effective reproduction number.

Our findings are consistent with a previous study [26] that showed a positive correlation of mobility and transmissibility of SARS-CoV-2 using different mobility datasets (from Apple and Google) and death counts. With a novel approach that uses Facebook mobility and case data, we estimated expected effective reproduction numbers and were able to show that transmissibility closely follows mobility, particularly when there is no evidence of ongoing local transmission (i.e., no reported cases). This supports the necessity of public compliance, which largely varied across the 10 WPRs. The reduction in mobility was significantly greater in WPRs with tighter, mandated movement restrictions (i.e., lockdown) including Singapore, New Zealand, Australia, Malaysia, Vietnam, and the Philippines compared to those with voluntary measures such as Taiwan; South Korea; Hong Kong; and Japan. While the heterogeneity in adherence to policies across WPRs is most likely due to differences in the type and scale of social distancing measures, and ongoing testing practices, and scale of contact tracing efforts, behavioural responses may also play a role. For example, previous studies have found that along with cultural differences, individual factors including socioeconomic status, perceptions of susceptibility or severity, self-efficacy, or trust in the government are significantly associated with the adoption of precautionary measures [2, 27]. According to recent cross-sectional studies conducted in the early stage of the pandemic, the perceived risk of contracting COVID-19 among healthy individuals largely varied between WPRs, from 20% for Korea [28], 31% for Malaysia [29], 47% in Singapore [30], and to 89% for Hong Kong [31].

In addition, we found a substantial difference in public compliance between urban and rural areas. The overall reduction in relative mobility was much more substantial in urban areas compared to provinces or rural areas in all WPRs and areas included in the analysis. Poor adherence to social distancing policies observed in some rural provinces, in which increased mean mobility was observed, could impede national efforts to contain the spread. Moreover, individuals living in rural and remote areas where health care capacity is often limited may be at higher risk of developing severe complications from COVID-19 [13], as they are more likely to be older and to have existing chronic conditions [10]. It should be noted however that differences in the strength of social distancing implementation may exist between rural and urban regions outside of differences in adherence.

Given that there were no variations in the level of social distancing measures implemented between urban and rural areas, the difference may be due to lower perceived susceptibility among individuals living in highly remote and rural areas where the number of new daily infections has remained relatively low throughout the COVID-19 pandemic. Another possible reason is that many individuals in remote areas need to go to larger cities on a regular basis for essential reasons such as to work, buy supplies, or receive medical treatment. It could also be associated with a high proportion of individuals working in agriculture, forestry, or fishing in rural areas for whom work-from-home is not a viable option. Additional research exploring factors and barriers affecting behavioural responses in individuals living in urban and rural areas is essential for developing more tailored and effective strategies which could potentially increase public acceptance and compliance in rural areas.

This study is subject to several limitations. First, since the mobility data used in the analysis were based on the movement of Facebook users, it may not best capture the movement patterns of older or rural populations who are less likely to use social media. However, it should also be noted that social distancing orders such as school and workplace closure mostly affect younger and middle-aged individuals and that the effects of such measures in senior adults are expected to be relatively minimal during this period. Moreover, Facebook is used by all individuals regardless of demographics (e.g., age, gender, and income) and across all regions. While it is most popular among individuals aged between 18 and 34, there is also a good representation of older adults aged 55 and over, accounting for about 11% of all Facebook users [32]. Of 2.74 billion monthly active users, about 42% (1.17 billion) are from countries in WPRs despite it still being banned in mainland China. The same report identified the Philippines as one of Facebook's fastest growing countries with the rapid adoption of digital technologies across the region, including rural areas. While only the US data is available, another survey shows that Facebook is used by individuals across income ranges, making it the most popular social network in low-income households [33]. Secondly, the baseline period (i.e., February 2020) may not be representative in WPRs that saw the outbreak earlier than this time. For example, South Korea reported 2,337 cumulative cases by the end of February [7] where individuals may have already started voluntarily limiting their movement, which can be contrasted to New Zealand which reported the first cases in late March (Supplementary Figure 7). Nonetheless, the relative mobility data shows overall movement trends, which can reflect changes in perceived susceptibility and public acceptance to different levels of social distancing orders in South Korea over time. Lastly, our *R*_*t*_ estimates should be interpreted as a potential or an expected reproduction number rather than a true *R*_*t*_, particularly for cities or states with no evidence of ongoing community transmission. It is because when there are no or very few cases, there is not enough information for our model to make a reliable inference of the reproduction number.

In conclusion, this study shows that the effectiveness of COVID-19 social distancing policies in mitigating the impact largely depends on public compliance. Significant differences in behavioural responses between urban and rural areas highlight the importance of taking a systematic approach that incorporates demographic, cultural, environmental, and socioeconomic factors of the target population to promote a community-wide effort and adherence to social distancing policies. The lifting of restrictions must also be carefully planned and executed gradually in phases as small increases in mobility can potentially lead to the resurgence of infections, as evidenced by many WPRs.

## Figures and Tables

**Table 1:** Changes in relative mobility and time-varying reproduction number during social distancing (SD) or community-wide stay-at-home (SH) orders.

City/state	Intervention		Changes in mobility^∗^	Time-varying **R**_**t**_	
			Median (range)	Start^#^ (95% CrI)	End (95% CrI)
Hong Kong	SD	3/29–5/07	−0.02 (−0.27, +0.20)	1.72 (1.60–1.83)	1.10 (0.98–1.22)
Seoul	SD	3/21–4/19	−0.05 (−0.16, +0.08)	1.61 (1.47–1.74)	0.93 (0.81–1.06)
Tokyo	SD	4/08–5/25	−0.34 (−0.61, −0.07)	1.45 (1.37–1.52)	0.80 (0.72–0.89)
Victoria	SH	3/31–5/12	−0.39 (−0.73, −0.11)	1.41 (1.32–1.52)	1.19 (1.07–1.32)
Kuala Lumpur	SH	3/18–5/03	−0.67 (−0.72, −0.33)	2.01 (1.88–2.14)	1.12 (1.03–1.21)
Manila	SH	3/15–5/15	−0.69 (−0.79, −0.30)	2.50 (2.35–2.66)	1.08 (1.03–1.13)

^∗^Changes in mobility relative to a baseline (e.g., -0.02 indicates 2% reduction in mobility from February 2020). ^#^**R**_**t**_ estimate for (or closest to) the first day of the intervention.

## Data Availability

The statistical code and datasets are available from the corresponding author upon request.

## Supplementary Materials

Supplementary Materials Figure 1: Hong Kong, Singapore, and Taiwan A. Countries with social distancing orders Changes in mobility relative to baseline (February 2020) and the time-varying reproduction number *Rt*. Figure 2: Japan. Figure 3: South Korea B. Countries with Stay-at-Home orders Changes in mobility relative to baseline (February 2020) and the time-varying reproduction number *Rt*. Figure 4: Australia. Figure 5: Malaysia. Figure 6: The Philippines. Changes in mobility relative to baseline (February 2020). Figure 7: New Zealand. Figure 8: Vietnam.
