# Supplementary material for: Urban-Rural Disparities for COVID-19: Evidence from 10 Countries and Areas in the Western Pacific
Source: Health Data Sci. 2021 Jun 12;2021:9790275. doi: 10.34133/2021/9790275 (PMC9629684; doi:10.34133/2021/9790275)
Supplement: Supplementary Materials — Figure 1: Hong Kong, Singapore, and Taiwan A. Countries with social distancing orders Changes in mobility relative to baseline (February 2020) and the time-varying reproduction number Rt. Figure 2: Japan. Figure 3: South Korea B. Countries with Stay-at-Home orders Changes in mobility relative to baseline (February 2020) and the time-varying reproduction number Rt. Figure 4: Australia. Figure 5: Malaysia. Figure 6: The Philippines. Changes in mobility relative to baseline (February 2020). Figure 7: New Zealand. Figure 8: Vietnam. [file 9790275.f1.pdf]

Supplementary Information for:

Urban-rural disparities for COVID-19: Evidence from 10 countries and areas in the Western Pacific

Supplementary Figures

Figure 1: Hong Kong, Singapore, and Taiwan, China…………………………………………………………..…………..……....1

A. Countries with social distancing orders

Changes in mobility relative to baseline (February 2020) and the time-varying reproduction number R_t

Figure 2: Japan………………………………………………………………………………………….…………..…..…..…...2-3

Figure 3: South Korea………………………………………………………………………………………….……......4-5

B. Countries with Stay-at-Home orders

Changes in mobility relative to baseline (February 2020) and the time-varying reproduction number R_t

Figure 4: Australia…………………………………………………………………………………………..…...….......6

Figure 5: Malaysia………………………………………………………………………………………….…...……..7-8

Figure 6: The Philippines…………………………………………………………………………………..…………….9-10

Changes in mobility relative to baseline (February 2020)

Figure 7: New Zealand………………………………………………………………………………………….……11-12

Figure 8: Vietnam……………………………………………………………………………..………………...……..13

Hong Kong (SAR China), Singapore, and Taiwan, China

Hong Kong and Taiwan, China were once considered to have the highest risk of COVID-19 epidemic due to its proximity to mainland China. However, the rapid implementation of non-pharmaceutical interventions including border closures and mask wearing in the early stage of the pandemic has led to a successful control of COVID-19.

With a spike of infection among migrant workers living in dormitories, Singapore implemented a lockdown measures ("Circuit Breaker") in an effort to control the spread in local communities while conducting extensive testing in all foreign worker dorms since early April 2020. Mobility was substantially reduced during the intervention period both in Hong Kong and Singapore.

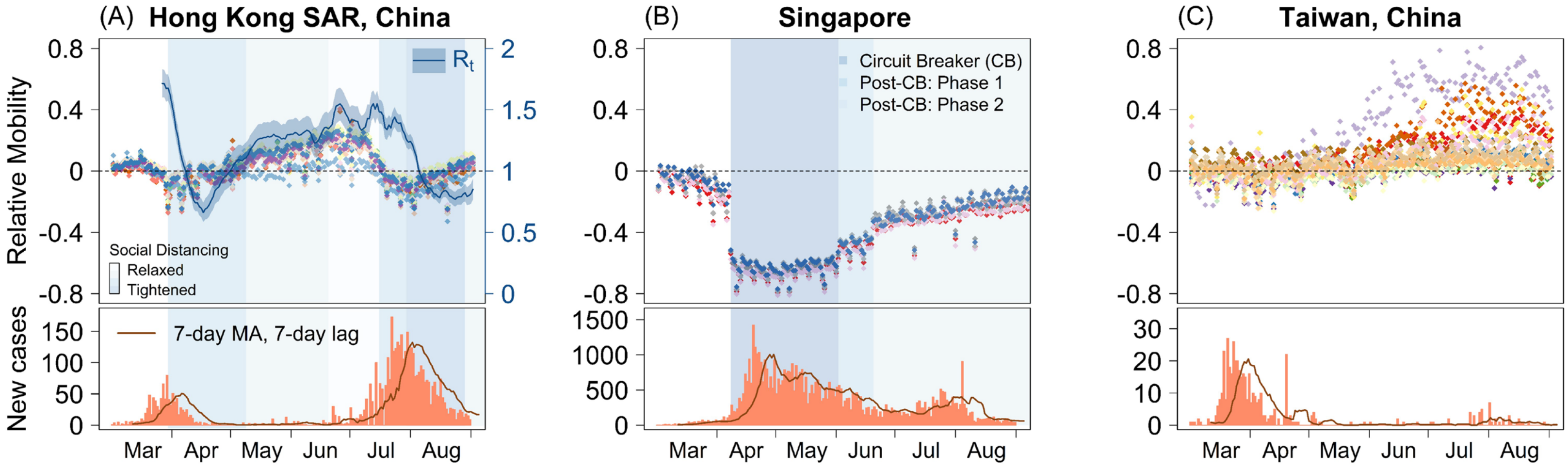

Supplementary Figure 1. Changes in relative movement between March 1 and August 31, 2020 in Hong Kong SAR China, Singapore, and Taiwan, China. Each color in the background indicates a different level of social distancing measures. The time-varying reproduction number was not estimated in Singapore as the vast majority of cases were reported in quarantined foreign worker dorms through aggressive routine testing and there were very few local cases. Similarly, there were not enough local cases to estimate the effective reproduction number in Taiwan, China. Province-level data were also not available at the time of the analysis.

Japan: Prefectures

A sharp reduction in relative mobility was observed in all 8 regions of Japan during the period in which “State of Emergency” was in place. The time-varying effective number dropped to below the threshold of 1 in all regions. Easing of restrictions coincided with an increased movement of the population (around the mobility threshold of 0) and a resurgence of infections in many regions.

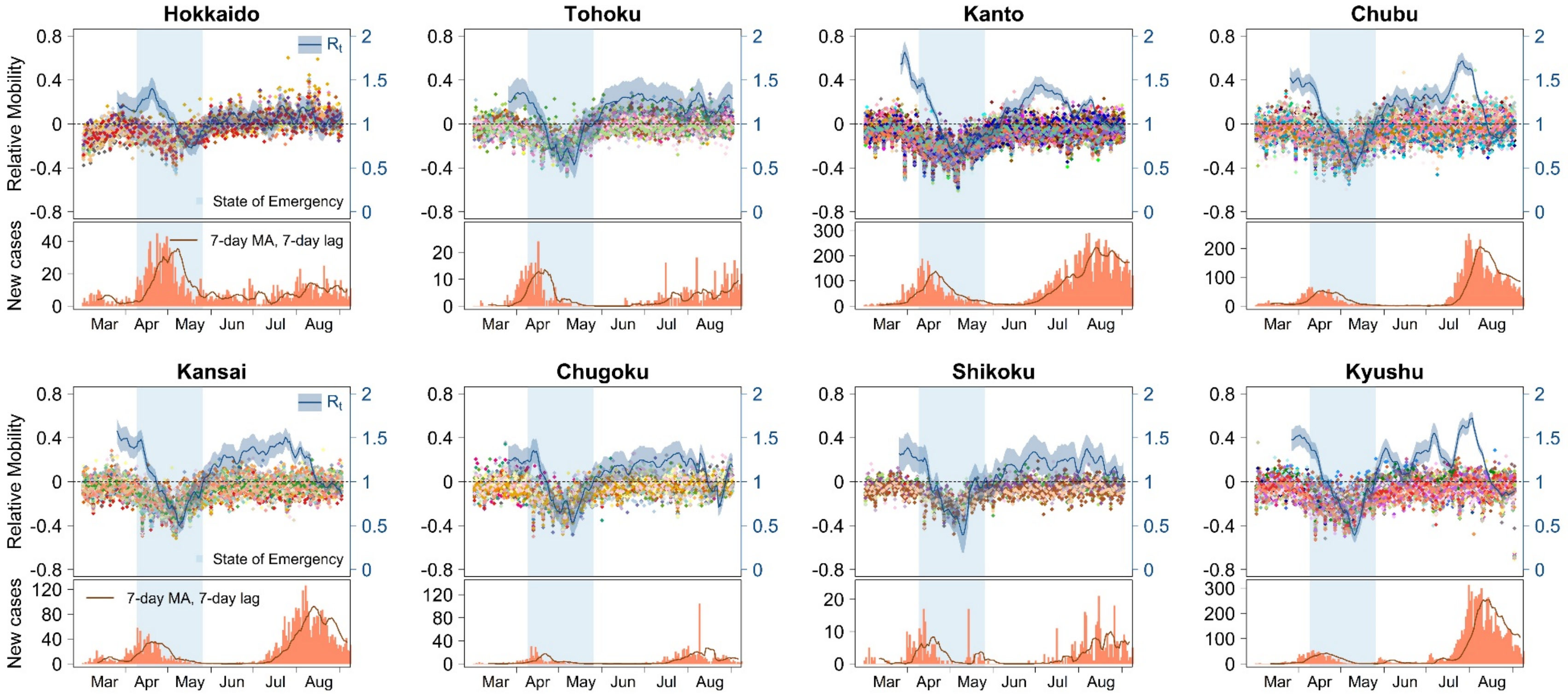

Supplementary Figure 2.1. Changes in relative movement and the time-varying reproduction number (Rt) between March 1 and August 31, 2020 in Japan, by region. Each color in the background indicates a different level of social distancing measures.

Japan: Metropolis and Urban Prefectures

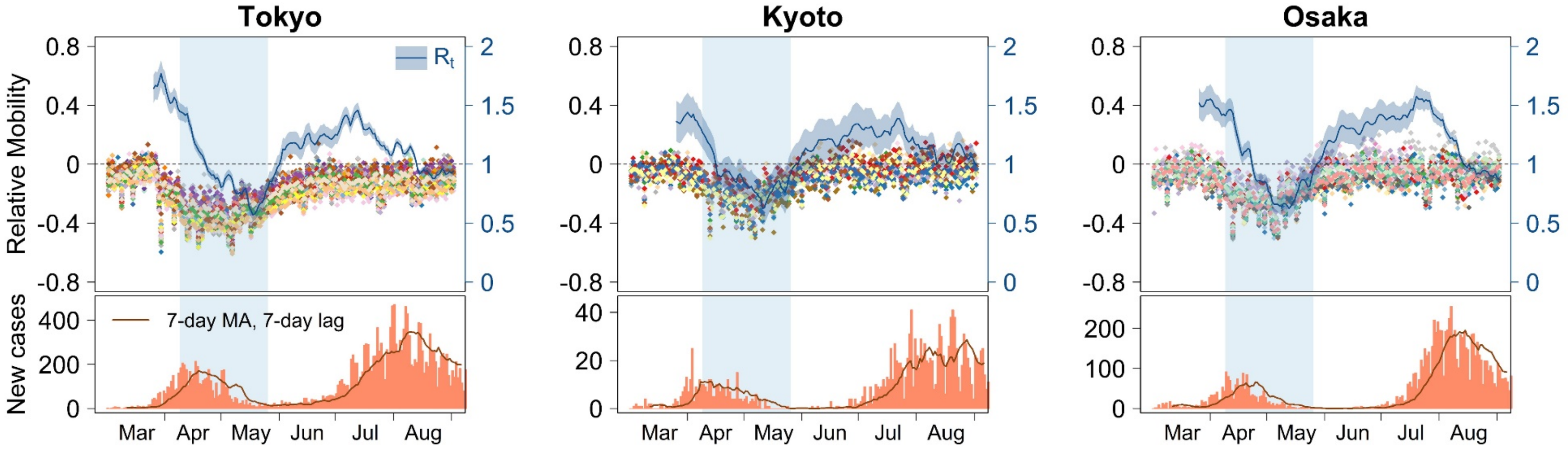

Supplementary Figure 2.2. Changes in relative movement and the time-varying reproduction number (Rt) between March 1 and August 31, 2020 in a metropolis (Tokyo) and urban prefectures (Kyoto and Osaka) of Japan. Each color in the background indicates a different level of social distancing measures.

South Korea: Metropolitan and Special Cities

Within South Korea, there was a significant difference in mobility patterns between metropolitan cities and rural provinces. Mobility gradually increased over time until the strictest social distancing measures were implemented in the end of August.

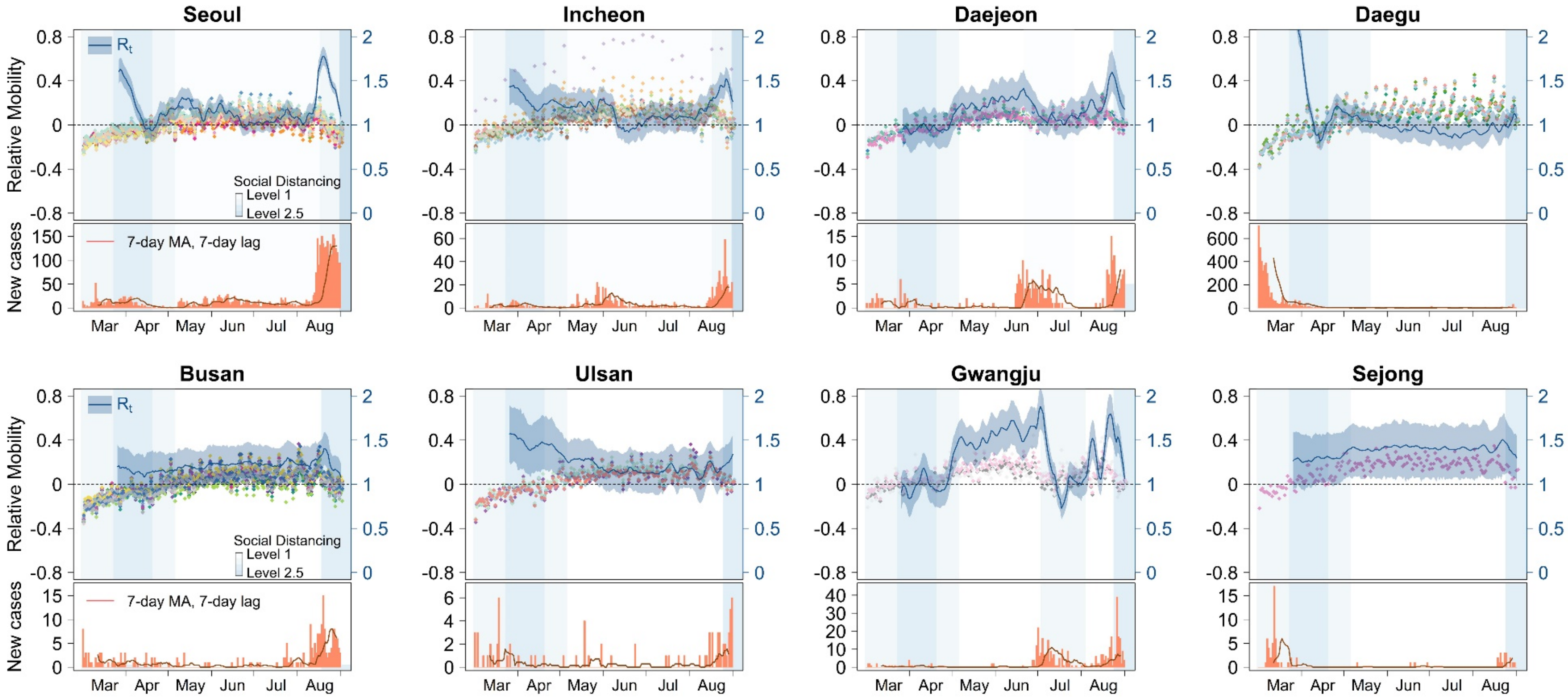

Supplementary Figure 3.1. Changes in relative movement and the time-varying reproduction number (Rt) between March 1 and August 31, 2020 in 8 metropolitan and special cities of Korea. Each color in the background indicates a different level of social distancing measures.

South Korea: Provinces

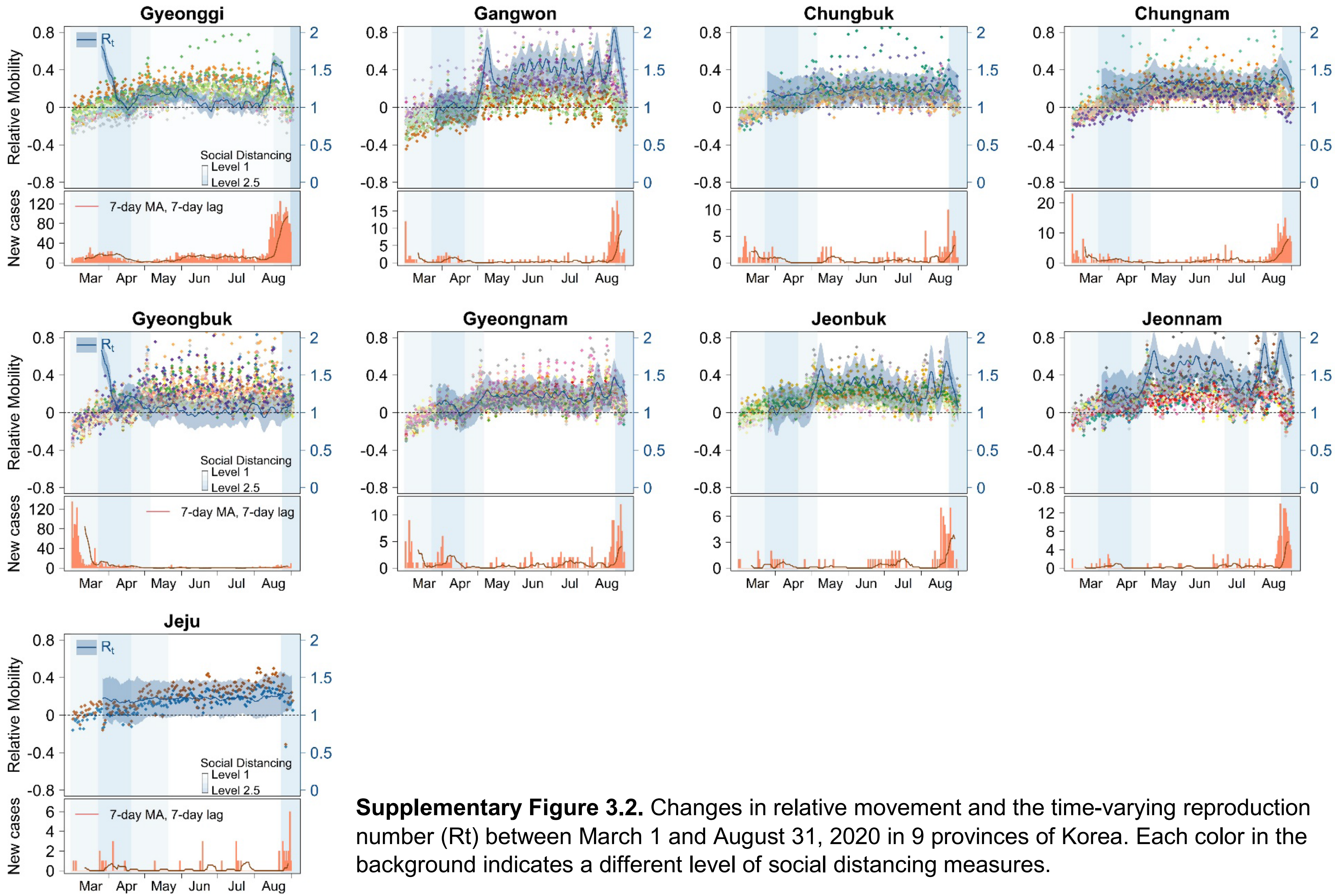

Supplementary Figure 3.2. Changes in relative movement and the time-varying reproduction number (Rt) between March 1 and August 31, 2020 in 9 provinces of Korea. Each color in the background indicates a different level of social distancing measures.

Australia

Strict lockdown measures were in place in most states of Australia by the end of March, which contributed to a drastic reduction in the potential reproduction number and subsequently the number of reported cases. Vitoria had a second wave of infections in July-August, placing Melbourne back into stage 4 lockdown and the regional Victoria to stage 3 measures following the declaration of a State of Disaster on Aug 2, 2020.

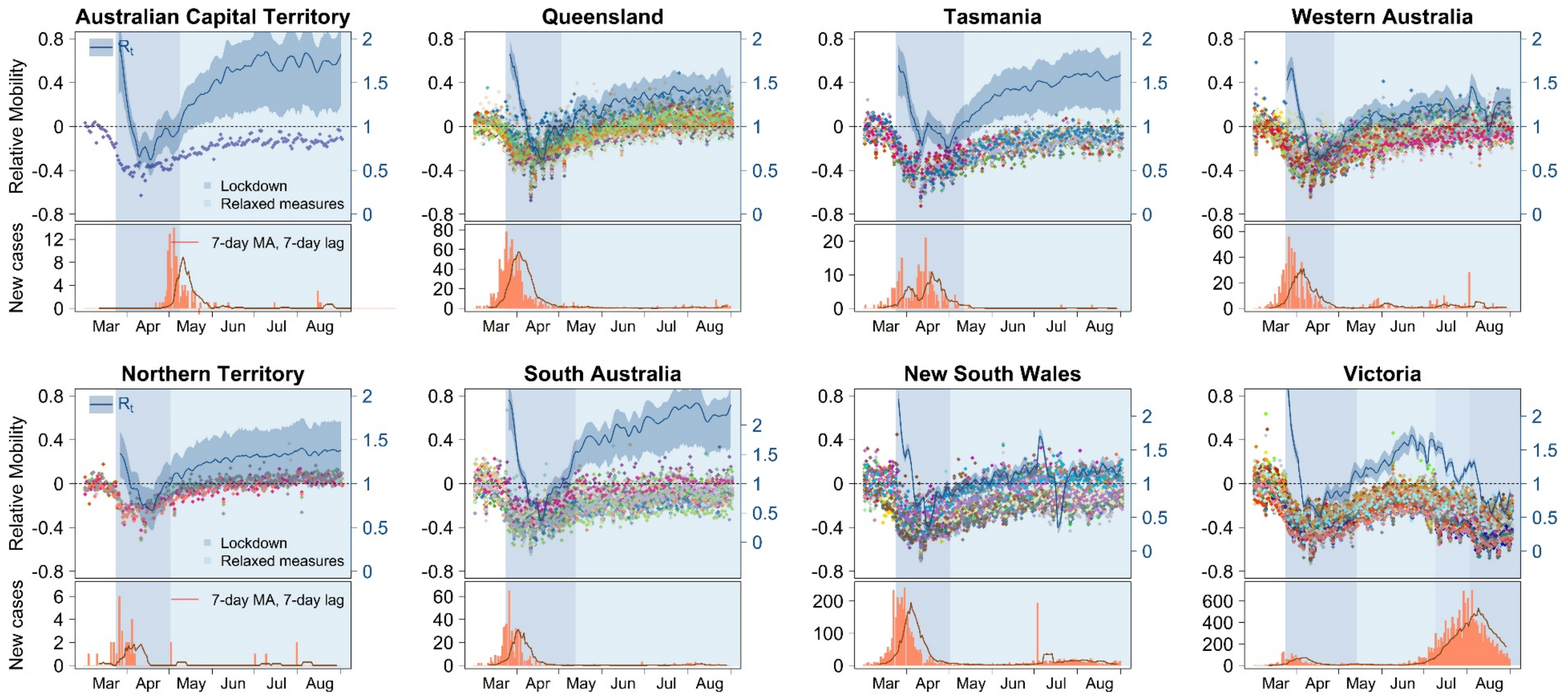

Supplementary Figure 4. Changes in relative movement and the time-varying reproduction number (Rt) between March 1 and August 31, 2020 in Australia, by state. Each color in the background indicates a different level of social distancing measures.

Malaysia

In response to an emergence of infections, a community-wide quarantine, namely "Movement Control Order (MCO)", involving the closure of all schools and non-essential businesses was implemented nationwide on March 18, 2020. This has led to a substantial reduction (<80%) in the overall mobility and the potential Rt in all states. As the government lifted several measures of the MCO starting from May, the movement gradually increased but the number of new infections remained low in the vast majority of cases by the end of August 2020.

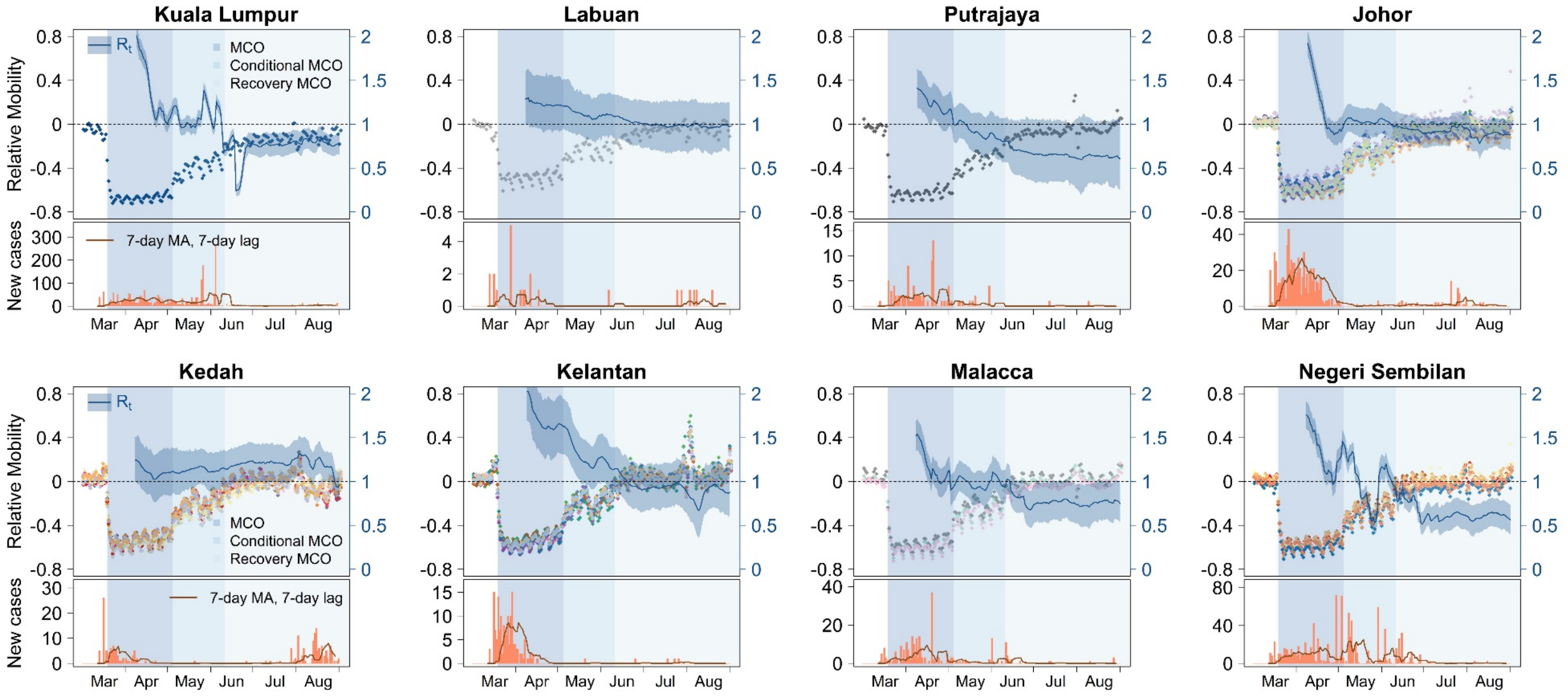

Supplementary Figure 5.1. Changes in relative movement and the time-varying reproduction number (Rt) between March 1 and August 31, 2020 in Malaysia, by state. Each color in the background indicates a different level of social distancing measures.

Malaysia (Cont'd)

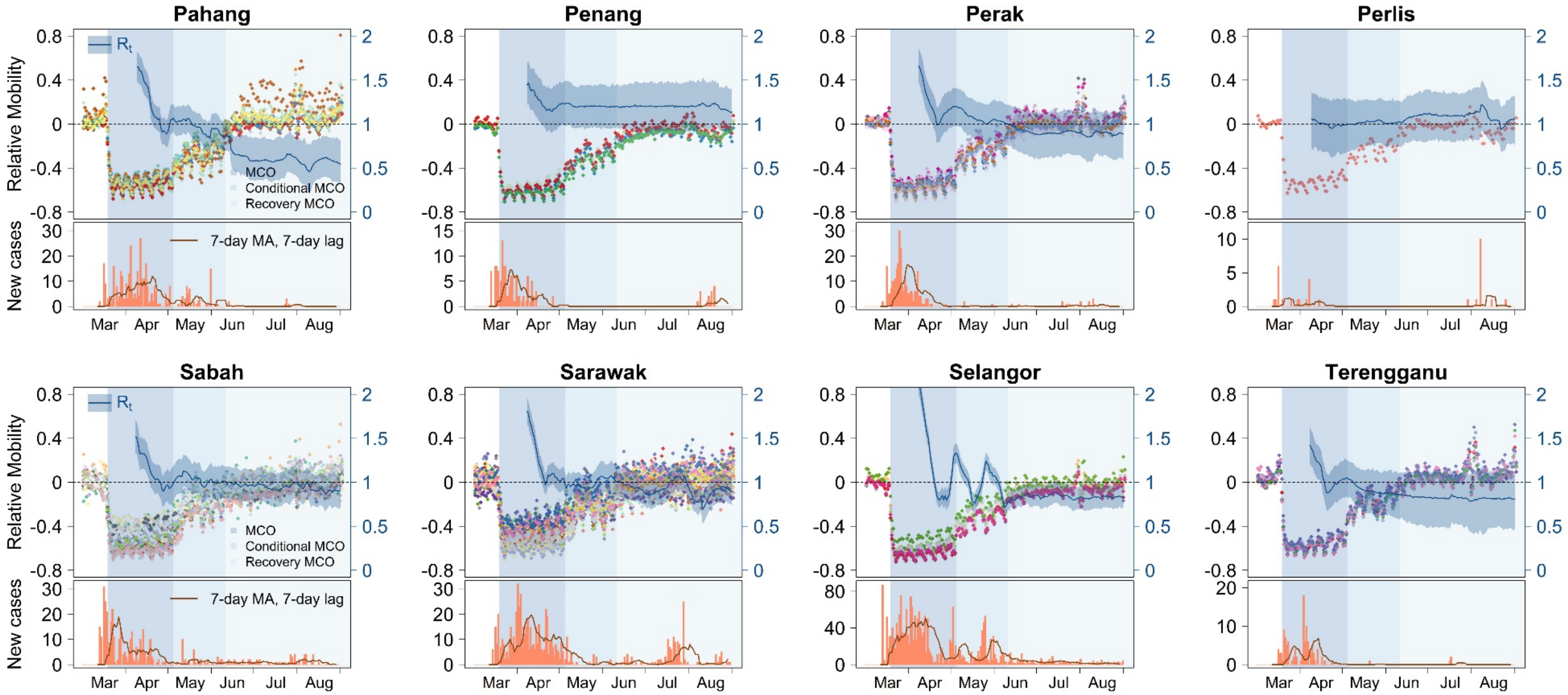

Supplementary Figure 5.2. Changes in relative movement and the time-varying reproduction number (Rt) between March 1 and August 31, 2020 in Malaysia, by state. Each color in the background indicates a different level of social distancing measures.

The Philippines

The Philippines implemented strict movement restriction policies early on with the emergence of COVID-19 in the Metropolitan Manila. The measures were effective in reducing mobility, up to 80% reduction compared to February 2020, and bringing down the potential Rt to close to the threshold of 1 in most regions of the Philippines. Gradual easing of restrictions, however, coincided with increase in mobility followed by the increase in the potential Rt, leading to an outbreak in all states across the Philippines in July-August 2020.

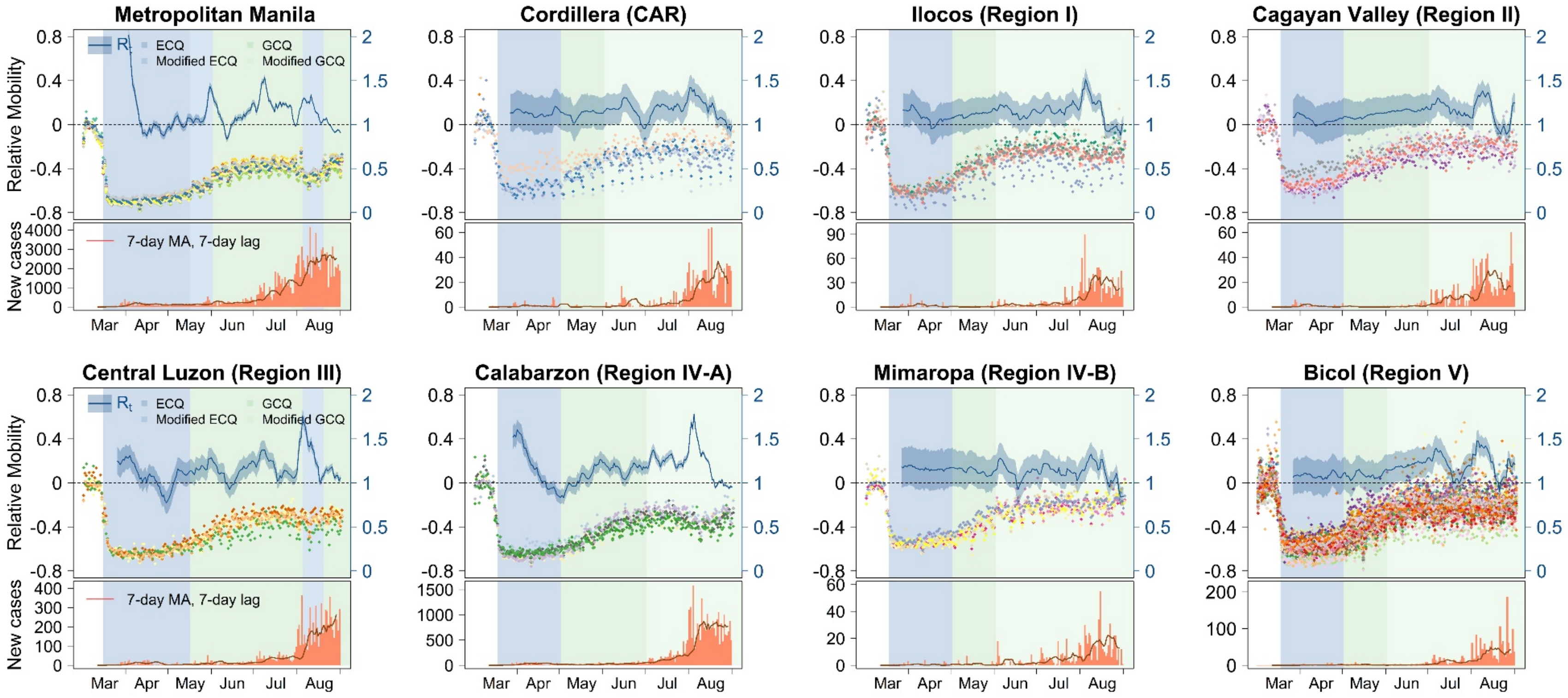

Supplementary Figure 6.1. Changes in relative movement and the time-varying reproduction number (Rt) between March 1 and August 31, 2020 in the Philippines, by region. Each color in the background indicates a different level of social distancing measures.

The Philippines (Cont'd)

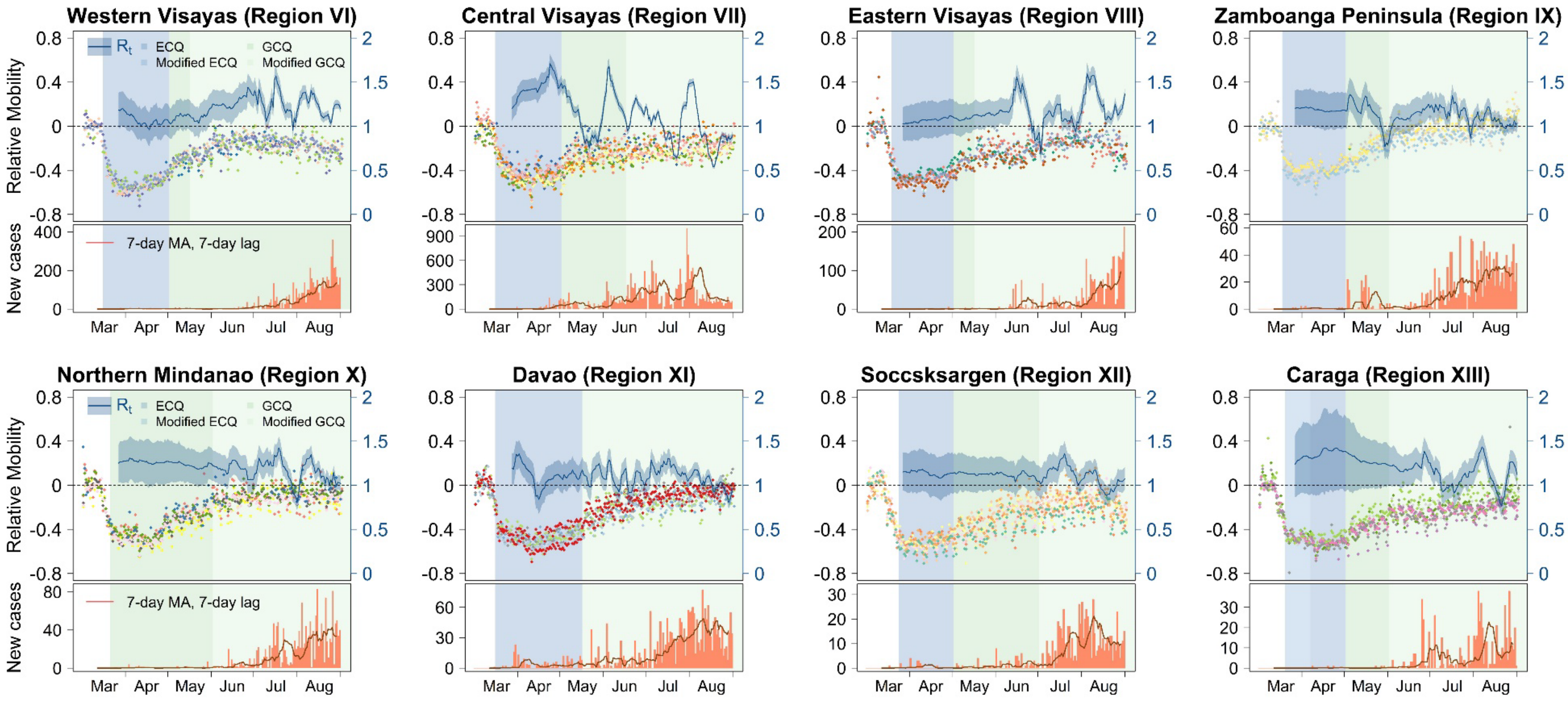

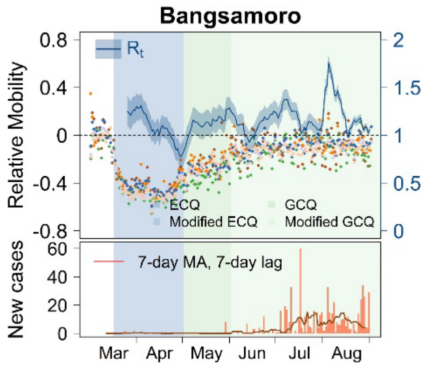

Supplementary Figure 6.2. Changes in relative movement and the time-varying reproduction number (Rt) between March 1 and August 31, 2020 in the Philippines, by region. Each color in the background indicates a different level of social distancing measures.

New Zealand

Compared to baseline period of February 2020, the movement of the population remained low (lower than the threshold mobility of 0) over the past six months of COVID-19 pandemic. In particular, mobility was reduced by about 70% in all regions of New Zealand during the nationwide lockdown period.

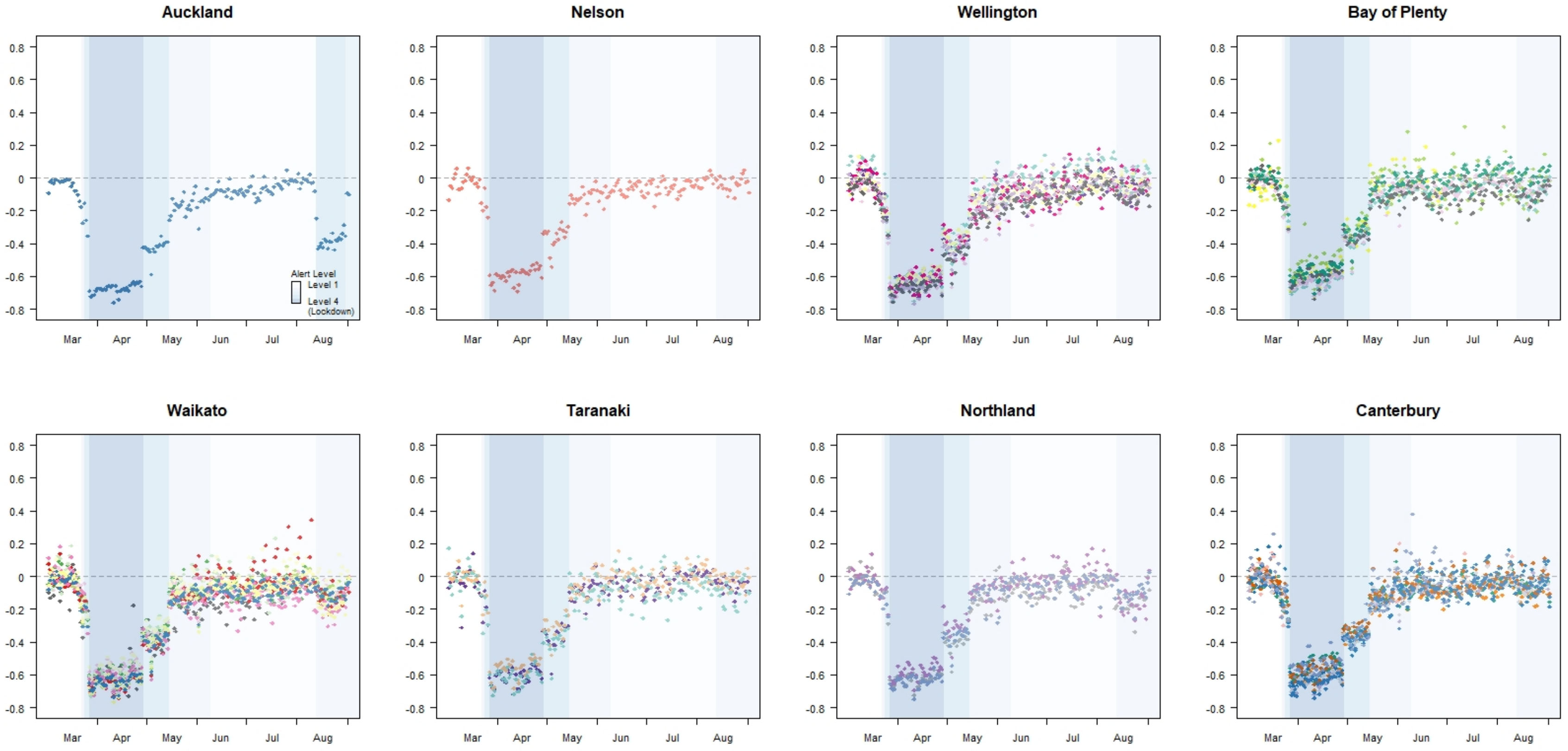

Supplementary Figure 7.1. Changes in relative movement between March 1 and August 31, 2020 in New Zealand, by region. Each color in the background indicates a different level of social distancing measures.

New Zealand (Cont'd)

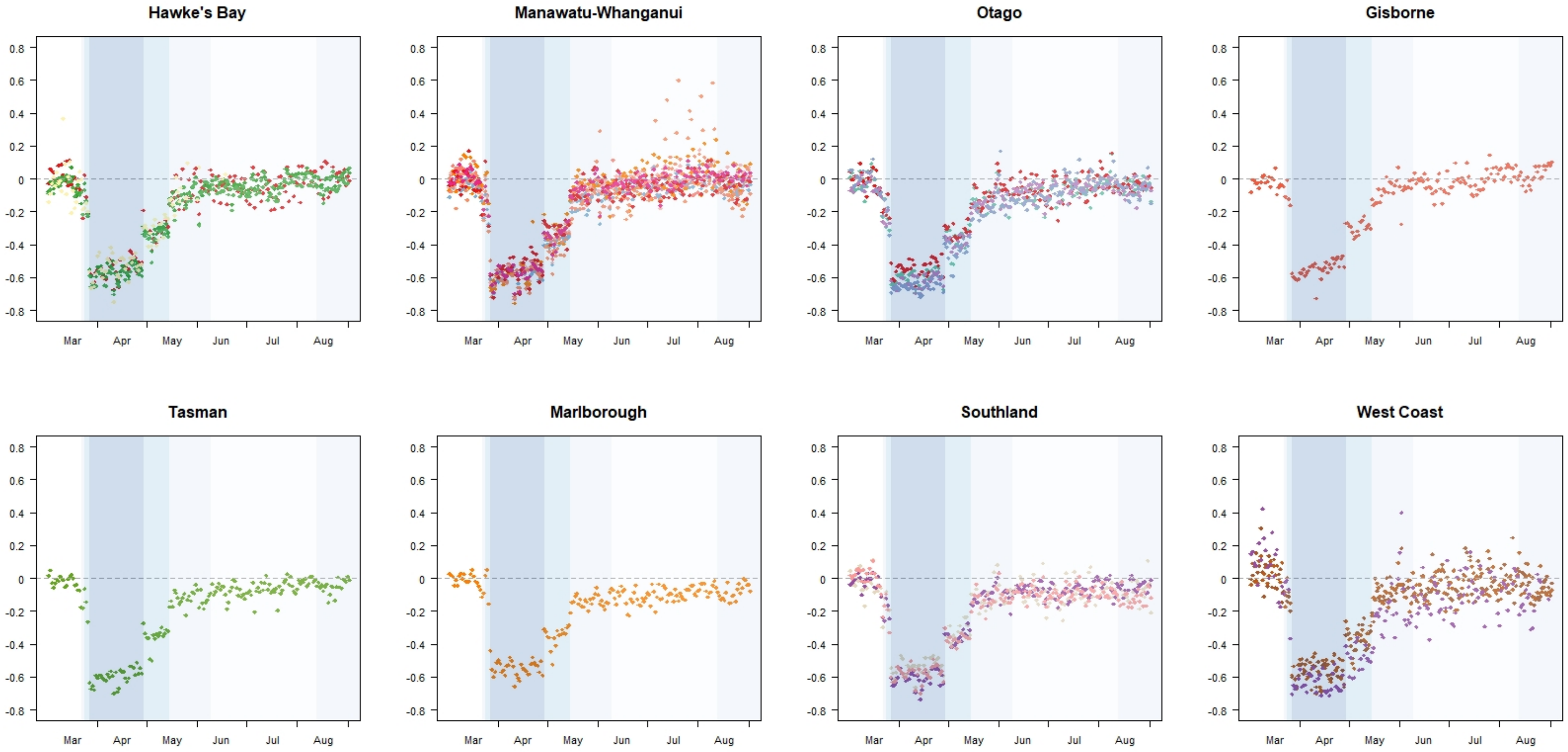

Supplementary Figure 7.2. Changes in relative movement between March 1 and August 31, 2020 in New Zealand, by region. Each color in the background indicates a different level of social distancing measures.

Vietnam

Overall, mobility remains similar to the baseline of February 2020 across eight regions in Vietnam. The nationwide lockdown imposed in early April reduced mobility to about 50%. Localized lockdown was implemented in several provinces in South Central Coast including Da Nang since late July following a spike in new cases in the region, which then reduced the overall mobility.

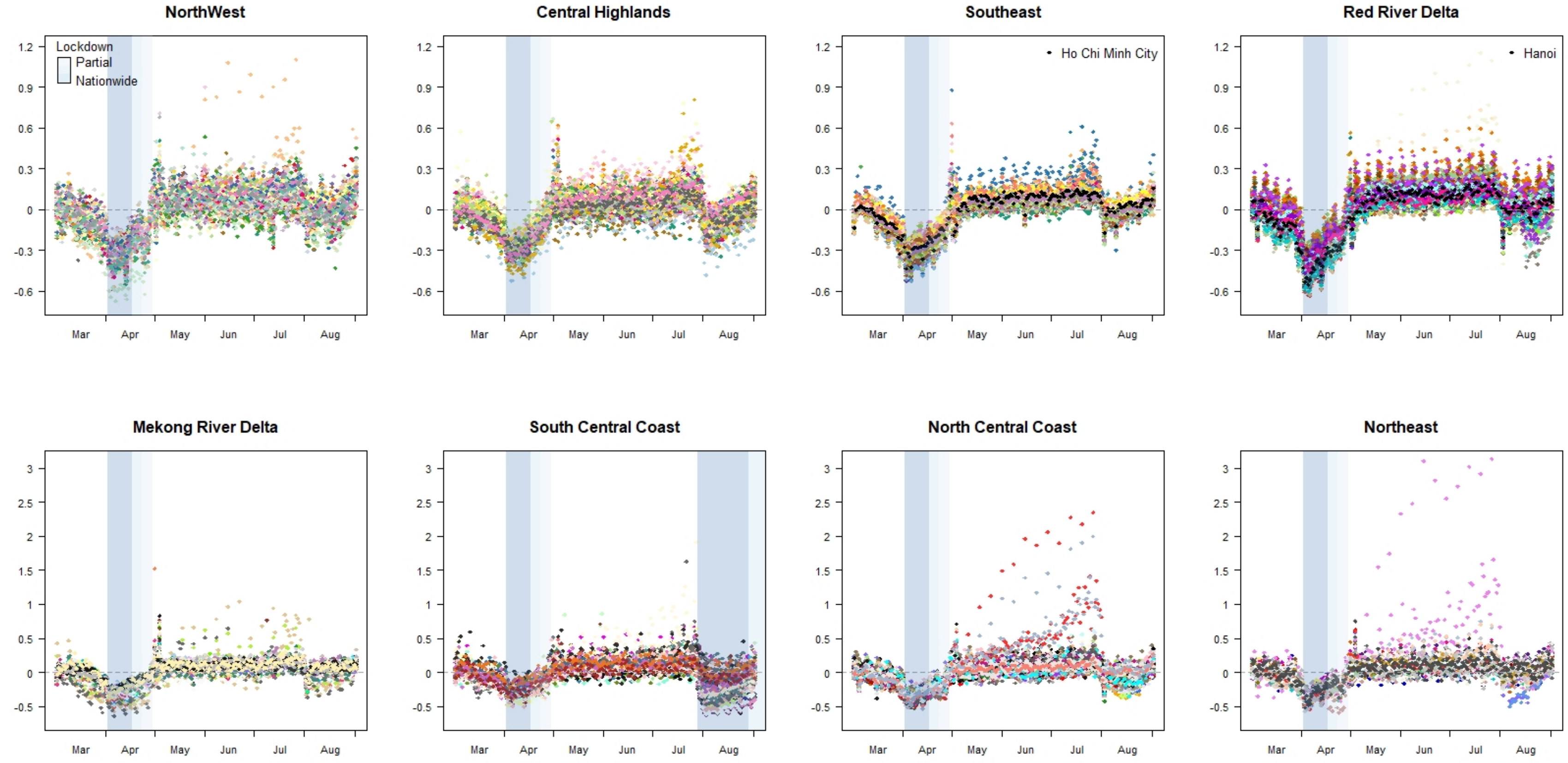

Supplementary Figure 8. Changes in relative movement between March 1 and August 31, 2020 in Vietnam, by region. Each color in the background indicates a different level of social distancing measures. Note: The Y-axis scale at top and bottom differs.
